# Changes in clinical migraine picture and in headache frequency in adolescence. Three year prospective study

**DOI:** 10.1186/1129-2377-1-S14-P220

**Published:** 2013-02-21

**Authors:** R Kalashyan

**Affiliations:** 1 Arabkir Medical Center, Armenia

## Background

The clinical picture of migraine in children was different from that in adolescents. The objective of the study was to examine factors which might contribute to an increase or decrease in headache frequency from the age of 13-16 years old. We compared the clinical findings at the first consultation at the age 13, and finally at the age of 16 years.

## Patients and methods

A population –based prospective study comprising a screening questionnaire at the age of 13 (n=717, response rate – 81%) by interview and clinical examination of randomly selected 182 children from migraine headache and adolescents with no headache. The patients followed in a Outpatients Pediatric Neurology Clinic between January 2009 December 2011. Finally, at the age of 16 years, 132 (73%)/182 adolescents could be re-examined. We checked the male/female ratio, side of the pain, nature of the pain, vertigo and/or dizziness and daily appearance of attacks.

## Results

The ratio of males was 43,2% and 34,1% in that order. Unilaterality of the headaches was 56,6 % and 73,2%, respectively. Pulsating quality was 36,4% and 47,3%. Vertigo and /or dizziness associated with headache were 10,2 % and 8,9% , and morning dominant attack cases 26,1% and 14,3%,respectively. Headaches increased clinically significantly in 22% (29% of girls and 14% of boys) and decreased in 17% (12% of girls and 23 % of boys).

## Conclusion

The present study revealed that the clinical picture of migraine in childhood and adolescents gradually changing to that for adults with aging. There are no reports on the correlation between the clinical picture and aging. Headache of migraine increases in girls, but decreases in boys in adolescence.

